# Resting-State Functional Connectivity in the Dorsal Attention Network Relates to Behavioral Performance in Spatial Attention Tasks and May Show Task-Related Adaptation

**DOI:** 10.3389/fnhum.2021.757128

**Published:** 2022-01-10

**Authors:** Björn Machner, Lara Braun, Jonathan Imholz, Philipp J. Koch, Thomas F. Münte, Christoph Helmchen, Andreas Sprenger

**Affiliations:** ^1^Department of Neurology, University Hospital Schleswig-Holstein, Campus Lübeck, Lübeck, Germany; ^2^Center of Brain, Behavior and Metabolism, University of Lübeck, Lübeck, Germany; ^3^Department of Psychology II, University of Lübeck, Lübeck, Germany

**Keywords:** functional connectivity, spatial attention, resting-state fMRI, attention network, behavior

## Abstract

Between-subject variability in cognitive performance has been related to inter-individual differences in functional brain networks. Targeting the dorsal attention network (DAN) we questioned (i) whether resting-state functional connectivity (FC) within the DAN can predict individual performance in spatial attention tasks and (ii) whether there is short-term adaptation of DAN-FC in response to task engagement. Twenty-seven participants first underwent resting-state fMRI (PRE run), they subsequently performed different tasks of spatial attention [including visual search (VS)] and immediately afterwards received another rs-fMRI (POST run). Intra- and inter-hemispheric FC between core hubs of the DAN, bilateral intraparietal sulcus (IPS) and frontal eye field (FEF), was analyzed and compared between PRE and POST. Furthermore, we investigated rs-fMRI-behavior correlations between the DAN-FC in PRE/POST and task performance parameters. The absolute DAN-FC did not change from PRE to POST. However, different significant rs-fMRI-behavior correlations were revealed for intra-/inter-hemispheric connections in the PRE and POST run. The stronger the FC between left FEF and IPS before task engagement, the better was the learning effect (improvement of reaction times) in VS (*r* = 0.521, *p* = 0.024). And the faster the VS (mean RT), the stronger was the FC between right FEF and IPS after task engagement (*r* = −0.502, *p* = 0.032). To conclude, DAN-FC relates to the individual performance in spatial attention tasks supporting the view of functional brain networks as priors for cognitive ability. Despite a high inter- and intra-individual stability of DAN-FC, the change of FC-behavior correlations after task performance possibly indicates task-related adaptation of the DAN, underlining that behavioral experiences may shape intrinsic brain activity. However, spontaneous state fluctuations of the DAN-FC over time cannot be fully ruled out as an alternative explanation.

## Introduction

Inter-individual differences in cognitive abilities have been related to inter-individual differences in functional brain networks (Baldassarre et al., 2012; Harmelech and Malach, 2013; Finn et al., 2015). These functional networks are considered to be shaped by lifelong learning experiences providing an indispensable memory system for upcoming cognitive challenges (Harmelech and Malach, 2013; Sadaghiani and Kleinschmidt, 2013). They can be assessed by analyzing spontaneous low-frequency fluctuations of the blood-oxygen level dependent (BOLD) signal in functional MRI, usually while the brain is at rest (Biswal et al., 1995). Remote brain regions of temporally coherent oscillations are regarded as functionally connected within one resting-state network (RSN) (Lowe et al., 1998; Fox and Raichle, 2007). RSNs comprise different functional domains, e.g., motor action, visual perception or attention, and the regions belonging to one RSN are also activated together when the brain is actively engaged in a related task (Smith et al., 2009).

While RSNs are reproducible across different subjects (Damoiseaux et al., 2006), their specific functional connectivity (FC) pattern appears to be unique and very stable in the individual subject, almost acting as an individual “fingerprint” (Finn et al., 2015; Osher et al., 2019). As it was first shown for the sensorimotor network, the strength of the FC largely accounts for the variability in behavioral responses, indicating the RSNs’ relevance for behavior (Fox et al., 2007). Moreover, the individual FC within (or between) RSNs has been shown to predict individual performance (or improvement/learning) in different cognitive tasks such as visual search (VS) (Chou et al., 2013; Bueicheku et al., 2020), audio-/visual perception (Hipp et al., 2011; Baldassarre et al., 2012; Sadaghiani et al., 2015; Berry et al., 2017) or mirror drawing (Manuel et al., 2018).

Despite the stability of RSNs, within- and between-network FCs can immediately change when a task is performed, i.e., when the brain changes its state from rest (“idling”) to action (Szczepanski et al., 2013; Spadone et al., 2015). Moreover, repetitive training or only one session of a novel task can induce persisting reorganization of RSNs, as it has been shown for different cognitive domains, including visual perception (Lewis et al., 2009; Urner et al., 2013; Guidotti et al., 2015; Sarabi et al., 2018), VS (Bueicheku et al., 2019), memory (Dresler et al., 2017), language (Waites et al., 2005), and visuo-motor skills (Manuel et al., 2018).

The current study focused on the dorsal attention network (DAN) as one of the RSNs (Fox et al., 2006; Hacker et al., 2013). The DAN is centered on bilateral regions in the frontal and parietal cortex, including the frontal eye field (FEF) and the intraparietal sulcus (IPS) (Fox et al., 2006; Corbetta and Shulman, 2011). These regions are recruited when attention is voluntarily shifted to spatial locations (Corbetta and Shulman, 2002) or task-relevant salient objects (Shulman et al., 2009) as well as during intentional visual exploration using eye movements (Corbetta et al., 1998). Experimental tasks that typically activate DAN regions include the Posner paradigm assessing covert orienting and reorienting (Posner and Petersen, 1990; Vossel et al., 2006; Doricchi et al., 2010), the Landmark task requiring spatial judgments (Milner et al., 1993; Fink et al., 2000; Revill et al., 2011) and VS paradigms (Corbetta et al., 1998; Nobre et al., 2003).

Pursuing the hypothesis that RSNs represent individual traits/priors of cognitive ability (Harmelech and Malach, 2013; Sadaghiani and Kleinschmidt, 2013; Spadone et al., 2015), the current study investigated whether pre-task resting-state FC in the DAN can predict individual behavioral performance in spatial attention tasks. Furthermore, as RSN’s are assumed to be malleable over short (to long) temporal scales in order to allow lifelong learning (Sadaghiani and Kleinschmidt, 2013), we questioned whether the DAN’s FC or FC-behavior relationship can already be changed by one training session of spatial attention tasks.

## Materials and Methods

### Participants

We recruited 29 healthy adult participants, most of whom were students at the University of Lübeck. The inclusion criteria encompassed right-handedness as tested by the Edinburgh Handedness Inventory (Oldfield, 1971), a normal or corrected-to-normal visual acuity and intact color vision as tested by the Ishihara’s Test (Kanehara & Co., Ltd., Tokyo, Japan). Exclusion criteria were a known neurological, psychiatric, or ophthalmological disease.

Before participation, written informed consent according to the Declaration of Helsinki and its later amendments was obtained from each participant. The study was approved by the local Ethics Committee of the University of Lübeck (14-189).

Two of the participants had to be excluded due to excessive head motion in the MRI scanner (see section “Materials and Methods” on quality control of rs-fMRI data), leaving 27 subjects [female: *n* = 16 (59%); mean age: 24.2 ± 0.8 years, range: 19–38 years] for final analysis.

### Stimuli and Tasks

Participants viewed the stimuli on a screen (NNL LCD monitor, NordicNeuroLab, Bergen, Norway; active TFT, 69.8 × 39.3 cm visual area), positioned on the front end of the MRI scanner, *via* a mirror that was attached to the head coil (eye-to-screen distance: 130 cm, i.e., visual area 30.1° × 17.2°). A keypad with buttons for the manual responses was fixed at the participant’s right thigh using hook-and-loop tape.

Three different tasks were presented, each addressing a specific subcomponent of spatial attention. The (I) Posner task tests covert reflexive shifts of attention (orienting and re-orienting) (Posner et al., 1984), the (II) Landmark task visuospatial judgments (Milner et al., 1993) and the (III) VS task (Machner et al., 2018) overt shifts of attention by exploratory eye movements. All these tasks are known to activate the bilateral FEF and IPS as core regions of the DAN (Corbetta et al., 1998; Fink et al., 2000; Doricchi et al., 2010).

For our experiment, the original *Posner* paradigm was adapted to the scanner environment in analogy to a previous study (Thiel et al., 2004). The basic display showed a dark-gray background with a light-gray diamond at the center and two light-gray square frames (size 2°), positioned on the horizontal meridian at either side with an eccentricity of 12° from the center. As an attentional cue, one side of the central diamond briefly (200 ms) lighted up. After a delay of either 150 or 400 ms (stimulus onset asynchrony), the target (a light gray asterisk) appeared for 100 ms within one of the two peripheral frames. The inter-trial interval varied between 1650 and 1900 ms. The participant was instructed to press the left button on the keypad with the index finger as quick as possible upon target appearance. One run of the Posner task included 48 valid (cue and target on the same side) and 12 invalid (target on the opposite side as indicated by the preceding cue) trials, corresponding to a cue validity of 80%, which allows assessment of endogenous attention shifting (Chica et al., 2011).

The *Landmark task* represents a “perceptive version” of the established line bisection task (Schenkenberg et al., 1980) but is better suited for the scanner environment (Fink et al., 2000). A white line (20° or 24° of visual angle) appeared on a dark-gray background. It was pre-bisected with a vertical dash either directly in the middle, i.e., with both parts of the line having the same length, or with a leftward or rightward shift of either 0.25° or 0.5°. The vertical dash bisecting the horizontal line was always aligned with the screen’s center so that the absolute spatial position of the bisection mark on the screen could not be used as a cue for the final spatial judgment of the line length (Ellison et al., 2004). The participants were asked to judge which half of the line was longer or whether both were equal in length. If the left part of the line was estimated to be longer, they should press the left button on a keypad with their right index finger. In case of a longer segment on the right, they should press a button with their fourth finger and in case of equal lengths of both halves of the line they used their middle finger. The line was presented for 300 ms and participants had a maximum time of 2000 ms to give their response by pressing the respective button. One run of the Landmark task consisted of 80 trials, 32 of which showed a correctly bisected line with both halves having the same length (“equal”), 24 trials with a longer left segment and 24 trials with a longer right segment.

**FIGURE 1:**
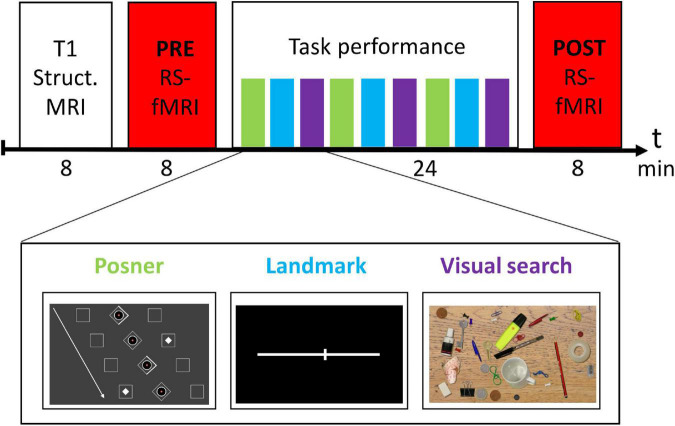
Experimental design.

In the *VS task* a computerized but naturalistic image of a desk scene was presented, in which participants were asked to find a paperclip (target) among different other everyday objects (e.g., a pen, coin, key, etc.) that served as distractors (Machner et al., 2018, 2020a). Each VS trial started with a central fixation cross presented on a black background, followed by the appearance of a desk image containing 30 different objects (see Figure 1 for an example). Participants were instructed to press a response button on a keypad as soon as they found the target. If there was no paperclip to be found, participants were asked to press a different button. A trial ended upon the button press or after a maximum time of 5000 ms. One run of the VS task included 30 trials of different desk images, 80% of which contained a paperclip (“target trials”).

### Experimental Design

The experimental design is depicted in Figure 1. Before entering the MRI scanner, subjects were briefly familiarized with the three tasks outside the MRI scanner. In the MRI scanner, subjects first received a structural MRI scan of the brain. Then, the PRE resting-state fMRI run was conducted. Next, there was the task block (total duration 24 min), in which participants performed three runs of each task in a predefined order (see Figure 1). Afterwards, the session was completed with a second rs-fMRI (POST) run.

To control for adherence to the tasks as well as for wakefulness during the rs-fMRI runs (eyes open), we continuously recorded and monitored eye movements of the participants using an MRI-compatible, remote eye tracker with a sampling rate at 1000 Hz (Eyelink 1000 Plus, SR Research, Ottawa, ON, Canada). This monitoring could exclude that participants fall asleep during the rs-fMRI sessions, also proven by offline analyses of eye position signals that showed only a small number of lacking eye signals in the PRE (14 ± 2%) and POST (18 ± 3%) rs-fMRI run, which were largely due to transient lid closure and artifacts.

### Behavioral Analysis

From the Posner task, we analyzed the mean reaction time (“RT mean”) of the responses in all the valid and invalid trials. Furthermore, we calculated the difference in RT between the invalid and valid trials (“RT invalid-valid”) as a more specific indicator for attentional reorienting (Rengachary et al., 2011). From the VS task, we analyzed the mean RT for trials, in which a target was present and the response button was correctly pressed. The performance in the Landmark task was assessed by calculating the “error rate (ER).” Therefore, the number of trials incorrectly judged was divided by the total number of trials presented ×100.

For each task, we additionally assessed the individual improvement by calculating the difference in the subject’s RT (ER, respectively) between the first and the last (third) run.

### Structural and Functional Magnetic Resonance Imaging

#### Acquisition of Imaging Data

Structural and functional MR imaging was performed at the CBBM Core Facility Magnetic Resonance Imaging using a 3-T Siemens Magnetom Skyra scanner equipped with a 64-channel head-coil. First, structural images of the whole brain using a 3D T1-weighted MP-RAGE sequence were acquired (TR = 2300 ms; TE = 2.43 ms; TI = 1100 ms; flip angle 8°; 0.85 mm × 0.75 mm × 0.75 mm resolution; 185 mm × 240 mm × 240 mm field of view; acquisition time: 8 min).

The resting-state functional image recordings were acquired by applying a single-shot gradient-recalled echo-planar imaging (GRE-EPI) sequence sensitive to blood oxygen level dependent (BOLD) contrast (480 volumes, TR = 1000 ms; TE = 30 ms; flip angle = 60°; in-plane resolution 3 mm × 3 mm; 204 mm × 204 mm field of view; 56 axial slices; 3 mm slice thickness, no interslice gap; simultaneous multi-slice factor 4; acquisition time: 8 min).

Lights were switched off during recordings. Subjects were asked to keep their eyes open and to foveate a small red dot on a black background. In order to minimize noise, ear plugs were used. Head movements were reduced by using ear pads (Multipad Ear, Pearltec Technology AG, Schlieren/CH).

#### Preprocessing of Resting-State fMRI Data

Preprocessing was performed using the DPARSFA toolbox (data processing assistant for resting-state fMRI, version 4.4^1^; Yan et al., 2016), while slice timing correction and further statistical analysis was performed with the SPM12 software^2^ (Wellcome Trust Centre for Neuroimaging, London, United Kingdom), both implemented in Matlab^®^ 2018B (MathWorks^®^, Natick, MA, United States).

First, the first 10 time points of each data set were discarded to allow for magnetization equilibrium and for subjects to adjust to the environment. The next steps included: (i) correction for differences in the image acquisition time between slices; (ii) a six parameter rigid body spatial transformation to correct for head motion during data acquisition; (iii) co-registration of the structural image to the mean functional image; (iv) gray and white matter segmentation, bias correction and spatial normalization of the structural image to a standard template (Montreal Neurological Institute, MNI); (v) regression of nuisance variables from the data (including white matter and ventricular signals, the six motion parameters determined in the realignment procedure as well as their first derivatives, the constant and linear trend); (vi) spatial normalization of the functional images using the DARTEL (Diffeomorphic Anatomical Registration Through Exponentiated Lie) method and resampling to 3-mm isotropic voxels; (vii) spatial smoothing with a 6 mm full-width at half-maximum Gaussian kernel. (viii) Before the FC analyses a temporal bandpass filter was applied to all voxel time series retaining only the low frequency spectrum (0.01–0.08 Hz).

#### Quality Control of Resting-State fMRI Data and Exclusion Due to Head Motion

The six realignment parameters, i.e., three displacements and three elementary rotations with respect to the first image in the EPI series, were used as an estimator for head motion. The maximum displacements were required to be smaller than 3.0 mm and individual rotations smaller than 3.0°. Because instantaneous motion below this threshold can still have a major confounding effect on rs-fMRI measures (Power et al., 2012; Van Dijk et al., 2012), we enabled DPARSFA to scrub the data by identifying and cutting out single motion contaminated frames (“bad” time points). The method is based on calculating the frame-to-frame displacement as described by Power et al. (2012), defining a “bad” time point when the framewise displacement threshold of >0.5 mm was exceeded and deleting the current time point (EPI volume), the previous one and the following two. The reduction of EPI volumes due to this cutting was required to be less than 38% in order to leave at least 5 min of rs-fMRI data for final analysis. Two subjects, who exceeded the cut-offs mentioned above, were excluded from final analysis. The remaining subjects had in the PRE rs-fMRI session on average a maximal head motion of 0.7 mm [standard deviation (SD): 0.4 mm], a mean framewise displacement of 0.2 mm (SD 0.1 mm), and for final analysis a mean reduction of 5.5% (SD 9.0%) EPI volumes due to the predefined cut-off of 0.5 mm framewise displacement. In the POST rs-fMRI session, their maximal head motion was on average 1.0 mm (SD 0.9 mm), the mean framewise displacement was of 0.2 mm (SD 0.1 mm), and the cut-off defined reduction of EPI volumes in the final analysis was 6.8% (SD 8.7%).

#### Definition of Regions of Interest

We defined the following regions of interest (ROIs) previously shown to be the most relevant hubs of the DAN (Fox et al., 2006; Corbetta and Shulman, 2011): the bilateral FEF and IPS. The center coordinates for the ROIs were taken from the literature, based on previous resting-state and/or task-related fMRI studies investigating the DAN (He et al., 2007; Hacker et al., 2013; Machner et al., 2020b): right FEF (23, −8, 55; *x*, *y*, *z* in MNI space], left FEF (−19, −8, 57) right IPS (27, −63, 54), and left IPS (−24, −60, 54). A 6 mm radius sphere was centered on the respective ROI coordinate, resulting in a ROI volume of ∼0.9 cm^3^ each.

#### Functional Connectivity Analyses

For the *seed-based (ROI-to-voxel) FC analyses*, the time courses of all voxels within a sphere ROI were averaged and then correlated to the time course of all the other voxels in the brain. The resulting connectivity maps of each participant were Fisher’s r-to-z transformed to obtain normally distributed measures for the subsequent statistical analyses. Next, group-wise ROI-to-voxel FC maps for each ROI and each rs-fMRI run were generated by calculating one-sample *t*-test contrasts that were corrected for multiple comparisons by applying the family-wise error (FWE) rate at the voxel level (corrected *p* < 0.05). Differences in ROI-to-voxel FC between the two rs-fMRI runs were assessed by calculating two-sample paired *t*-test contrasts (POST > PRE) for each ROI with the cluster-defining threshold set at *p* < 0.001 (uncorrected), followed by a *p* < 0.05 FWE correction at cluster level.

For the *pairwise (ROI-to-ROI) FC analyses*, the time courses of all voxels within a sphere ROI were averaged and correlated to the mean time course of voxels in the other sphere ROI. The resulting Pearson correlation coefficients of the four predefined intra-/interhemispheric ROI pairs (FEF_L_–FEF_R_, IPS_L_–IPS_R_, FEF_L_–IPS_L_, and FEF_R_–IPS_R_) were Fisher-z-transformed before entering further analyses.

For each participant, we also calculated one mean FC value for the whole DAN network (“DAN-FC”) in the PRE and the POST session by summing up the individual FC values of the predefined ROI pairs and dividing it by their number (*n* = 4).

### Statistics

Statistical analyses, apart from the fMRI analyses described above, were performed using the SPSS software package (version 22.0.0.2; IBM Corp., Somer, NY, United States).

Unless otherwise reported, data in the manuscript are presented as mean ± standard error of the mean (SEM). Differences in the ROI-to-ROI FC values between the PRE and the POST rs-fMRI run were assessed using paired *t*-tests. Correlation analyses between rs-fMRI (ROI-to-ROI FC) and behavioral (RT and ER, respectively) parameters were performed using the non-parametric Spearman’s rho correlation coefficient. The results of the rs-fMRI-behavior analyses, which tested whether behavioral performance was correlated to the DAN-FC in a hypothesis-driven set of four predefined ROI pairs, were corrected for multiple comparisons by applying the Bonferroni–Holm correction (*p*-value × 4). Furthermore, significant FC-behavior correlations in one rs-fMRI session (e.g., PRE) were tested for a significant difference to the corresponding rs-fMRI session (e.g., POST). Therefore we assessed the interaction FC × behavior by weighting the individual FC value with the linear regression coefficient from the FC-behavior correlation and subsequently performed a paired *t*-test on the weighted FC between PRE and POST. Regression coefficients were calculated using “robustfit” function within Matlab (DuMouchel and O’Brian, 1989). This function uses iteratively reweighted least squares to compute the coefficients, which makes it robust against extreme values and outliers. The level of significance was set at *p* < 0.05.

## Results

### Behavioral Task Performance

The participants’ mean RT in the Posner task was 435 ± 11 ms and there was significant improvement from the first to the last run (*d* = −32 ± 6, *p* < 0.001). The validity effect, i.e., the difference in RT between invalid and valid trials, was on average 32 ms ± 6. The mean RT in the VS task was 1500 ± 54 ms, again with significant improvement over time (*d* = −270 ± 49, *p* < 0.001). The mean ER in the Landmark task was 39 ± 1%. In this task, the participants revealed no improvement between the first and the last run (*d* = −1 ± 1%, *p* = 0.522).

### Resting-State fMRI Results

**FIGURE 2:**
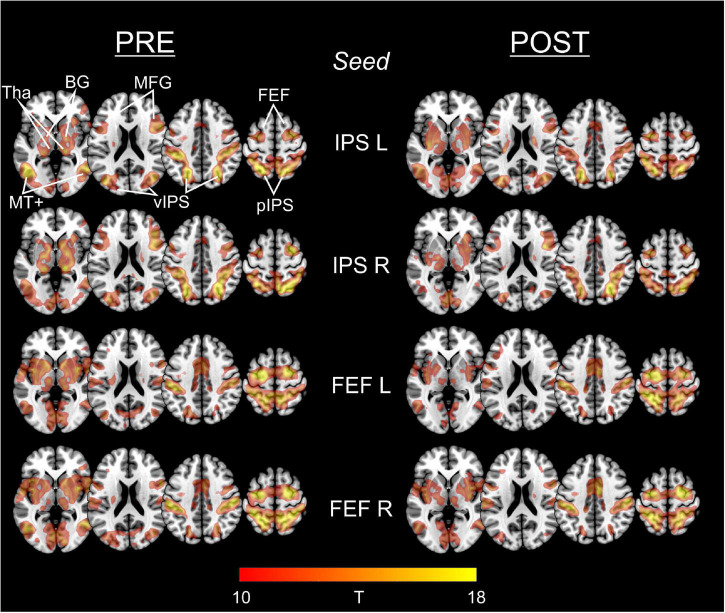
Functional connectivity of IPS and FEF seed regions in PRE and POST run. For each of the predefined DAN-ROIs (bilateral IPS and FEF) and separately for the rs-fMRI run before (PRE) and after (POST) task performance, group statistical FC maps were obtained using one-sample *t*-tests on the individual Fisher’s z-transformed correlation maps, corrected for multiple comparisons applying a false family-wise error (FWE) at *p* < 0.05. Results are presented at a threshold of *T* > 9.86 (equals FWE-corrected *p* < 0.00001), depicted on axial slices (*z*: 0, 30, and 60) of a MNI brain template. The predefined seed-ROIs almost uniformly show functional connections to other DAN regions including ventral/posterior IPS and FEF, middle frontal gyrus (MFG), middle temporal complex (MT+) as well as to the basal ganglia (BG) and thalamus (Tha).

Figure 2 depicts the statistical maps showing the results of the seed-based (ROI-to-voxel) FC analysis of the four predefined DAN-ROIs, separately for the PRE and POST rs-fMRI run.

For each of the four seeds, we consistently observed functional connections to the (bilateral) FEF, IPS, middle frontal gyrus (MFG), and MT+ (middle temporal complex) as well as to the basal ganglia and the thalamus.

Statistical comparison of the different seed-based FC maps between the PRE and the POST run did not reveal supra-threshold voxels. Hence, FC of the predefined ROIs to whole brain did not change significantly from PRE to POST.

Table 1 depicts the group-mean of Fisher’s z-transformed ROI-to-ROI FC results of the different intra- and inter-hemispheric ROI pairs, separately for the PRE and POST rs-fMRI run. Pairwise comparisons did not reveal significant differences between the PRE and POST rs-fMRI run (*p* always > 0.104).

**TABLE 1:** Intra- and interhemispheric ROI-to-ROI functional connectivity in the DAN before (PRE) and after (POST) task performance.

ROI pairs	z-FC in PRE	z-FC in POST	Statistics
**Inter-hemispheric**			
FEF_L_–FEF_R_	1.00 (0.05)	0.98 (0.05)	n.s.
IPS_L_–IPS_R_	0.61 (0.05)	0.52 (0.06)	n.s.
**Intra-hemispheric**			
FEF_L_–IPS_L_	0.38 (0.04)	0.40 (0.05)	n.s.
FEF_R_–IPS_R_	0.69 (0.04)	0.69 (0.06)	n.s.
**Whole network**			
DAN	0.67 (0.03)	0.65 (0.04)	n.s.

*Data are Fisher-z-transformed FC value (SEM).*

*Statistical comparison between rs-fMRI runs were performed using paired t-tests; n.s., not significant (p > 0.05).*

### Correlation of Behavioral and fMRI Parameters

Correlation analyses were performed between behavioral results (mean RT and RT improvement in the Posner and VS task, ER in the Landmark task) and ROI pairs’ FC results in the PRE and the POST run (Table 2).

**TABLE 2:** Overview of all FC–behavior correlations.

ROI-to-ROI FC		FEF_L_–FEF_R_		FEF_L_–IPS_L_		FEF_R_–IPS_R_		IPS_R_–IPS_L_		DAN-FC	
Task performance	*Session*	*r*	*p*	*r*	*p*	*r*	*p*	*r*	*p*	*r*	*p*
Posner RT mean	*Pre*	0.114	0.573	0.043	0.832	0.108	0.592	–0.200	0.318	0.051	0.802
	*Post*	0.389	0.045	0.263	0.185	0.098	0.628	0.073	0.716	0.225	0.260
	*Post–Pre*	0.208	0.297	0.176	0.380	0.137	0.496	0.364	0.062	0.246	0.216
Posner RT change Run 1–3	*Pre*	**0.469**	**0.013**	0.354	0.070	–0.353	0.070	0.060	0.765	0.275	0.165
	*Post*	–0.299	0.130	–0.224	0.261	**−0.676**	**0.000**	–0.440	0.022	**−0.581**	**0.001**
	*Post–Pre*	−**0.635**	**0.000**	**−0.563**	**0.002**	–0.394	0.042	–0.379	0.051	**−0.651**	**0.000**
Posner RT invalid–valid	*Pre*	0.288	0.145	–0.123	0.542	–0.235	0.238	–0.325	0.098	–0.214	0.285
	*Post*	0.287	0.147	–0.210	0.293	–0.371	0.057	–0.370	0.058	–0.238	0.232
	*Post–Pre*	0.072	0.721	0.030	0.882	–0.256	0.197	–0.125	0.534	–0.059	0.769
Posner RT invalid–valid	*Pre*	–0.116	0.565	0.121	0.548	0.355	0.069	–0.090	0.654	–0.023	0.909
change Run 1–3	*Post*	0.026	0.897	0.335	0.087	0.189	0.344	–0.106	0.598	0.079	0.694
	*Post–Pre*	0.090	0.656	0.294	0.137	0.058	0.774	0.031	0.877	0.164	0.413
Visual Search RT mean	*Pre*	**0.527**	**0.005**	0.085	0.672	–0.364	0.062	–0.021	0.916	0.137	0.496
	*Post*	–0.017	0.933	–0.276	0.164	**−0.502**	**0.008**	–0.366	0.060	–0.415	0.031
	*Post–Pre*	–0.367	0.060	–0.393	0.042	–0.274	0.166	–0.358	0.066	–0.454	0.017
Visual search RT	*Pre*	0.288	0.146	**0.521**	**0.005**	–0.077	0.703	0.266	0.180	0.412	0.033
change Run 1–3	*Post*	0.070	0.730	0.139	0.491	–0.204	0.308	–0.185	0.356	–0.127	0.528
	*Post–Pre*	–0.101	0.617	–0.151	0.453	–0.119	0.554	–0.412	0.033	–0.281	0.155
Landmark ER mean	*Pre*	0.329	0.094	–0.002	0.992	0.401	0.038	–0.034	0.865	0.292	0.139
	*Post*	0.360	0.065	0.311	0.115	0.119	0.554	0.274	0.164	0.329	0.094
	*Post–Pre*	0.026	0.896	0.323	0.100	–0.281	0.156	0.238	0.233	0.094	0.640
Landmark ER change Run 1–3	*Pre*	0.140	0.485	0.036	0.859	–0.091	0.650	–0.031	0.880	0.023	0.911
	*Post*	0.047	0.814	0.159	0.428	–0.122	0.546	–0.080	0.690	–0.007	0.973
	*Post–Pre*	–0.131	0.514	0.046	0.820	–0.053	0.795	0.012	0.952	–0.051	0.800

*RT, reaction time; ER, error rate; “RT invalid-valid” difference in RT between invalid and valid trials; “change Run 1–3” difference between the first and third task run; DAN-FC, mean FC of all four ROI pairs.*

*p-Values are uncorrected. Bold values are significant at p < 0.05 after correction for multiple comparisons (see main text for details).*

**FIGURE 3:**
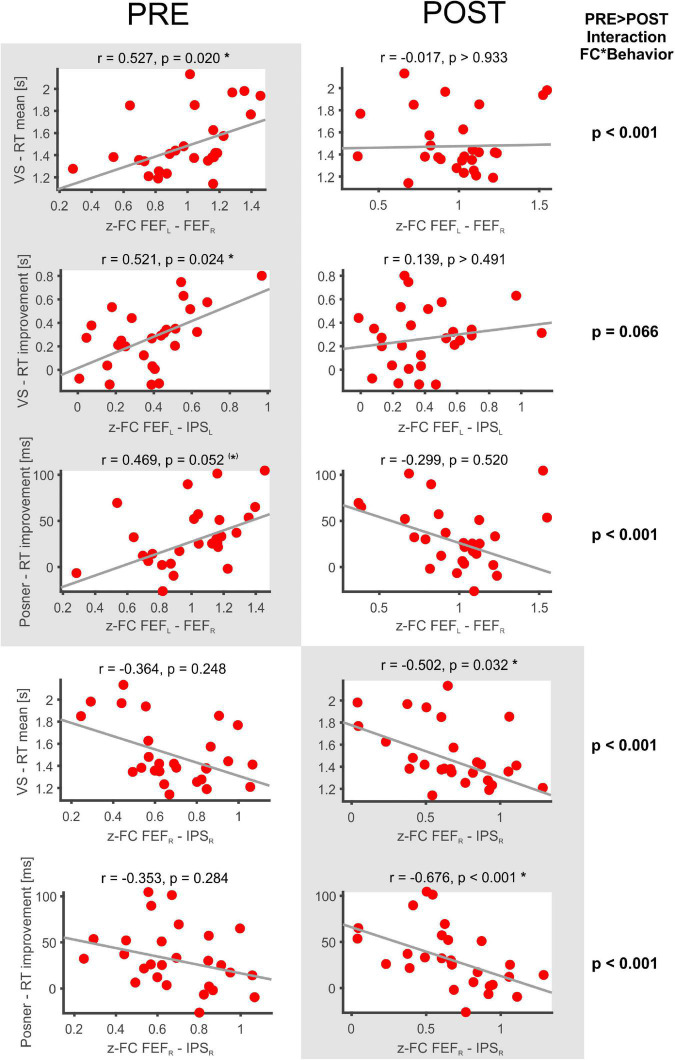
Significant FC-behavior correlations in the PRE and POST rs-fMRI session. The z-transformed FC of several DAN-ROI pairs is correlated with measures of behavioral performance in spatial attentions tasks (mean RT, RT improvement). For each correlation, the Spearman’s rank correlation coefficient (*r*) and a robust fit (gray line) is provided as well as the respective *p*-value (corrected for multiple comparisons applying Bonferroni–Holm). Significant correlations are highlighted on a gray background and marked with * (corrected *p* < 0.05) or (*) for a statistical trend (*p* < 0.1), while the non-significant counterparts in the PRE or POST condition are shown beside on a white background.

The following significant rs-fMRI-behavior correlations were revealed for the PRE run (Figure 3): The intra-hemispheric FC between left FEF and IPS correlated with the RT improvement in the VS task (*r* = 0.521, *p* = 0.024) as well as with the RT improvement in the Posner task (*r* = 0.496, *p* = 0.052, statistical trend). Thus, the stronger the participant’s FC between left FEF and IPS, the better was the individual learning effect in both tasks. Furthermore, the FC between right and left FEF was correlated with the mean RT in VS (*r* = 0.527, *p* = 0.020), i.e., participants with stronger interhemispheric FEF-FC needed on average more time to detect the target in the VS task.

In the POST run (Figure 3), the FC between right FEF and IPS was found to be inversely correlated with the mean RT in VS (*r* = −0.502, *p* = 0.032). Thus, participants with faster VS revealed stronger intrahemispheric FC in the right DAN after task performance. Moreover, the POST run FC between right FEF and IPS was negatively correlated with the RT improvement in the Posner task (−0.676, *p* < 0.001) and also the average DAN-FC was negatively correlated with the RT improvement in the Posner task (−0.581, *p* = 0.001). Hence, participants with greater improvement in the Posner task subsequently revealed more decreased intrahemispheric FC in the DAN.

When comparing these correlation results between PRE and POST, the interaction FC*behavior became significantly different in four (*p* < 0.001) and showed a statistical trend (*p* < 0.01) in one of the comparisons (Figure 3).

Finally, the individual difference in FC between the POST and PRE run was correlated to the different behavioral parameters (Table 2, POST-PRE lines). This yielded significant inverse correlations with the RT improvement in the Posner task for the two ROI pairs FEF_L_–FEF_R_ and FEF_L_–IPS_L_ as well as for the DAN-FC (Table 2, corrected *p* always < 0.05).

## Discussion

Using rs-fMRI, we investigated the FC of and between core regions of the DAN (FEF and IPS) *before* (PRE) and immediately *after* (POST) engagement in a set of spatial attention tasks (Posner, VS, Landmark). Beside the PRE-POST comparisons, we analyzed FC-behavior relationships for each rs-fMRI run, i.e., the respective correlation of ROI-to-ROI DAN-FC and behavioral measures of task performance. Based on the hypothesis that intrinsic functional networks determine individual cognitive abilities we expected that (i) DAN-FC strength could predict individual behavioral performance in spatial attention tasks. Furthermore, following the assumption that experiences shape functional brain networks, we expected (ii) DAN-FC to change from PRE to POST as well as the FC-behavior relationship to differ between PRE and POST.

### Dorsal Attention Network -Functional Connectivity Relates to Behavioral Performance in Spatial Attention Tasks

The strength of FC in several DAN-ROI pairs was found to be related to the behavioral performance in different tasks of spatial attention. For example, an increased FC between left-hemisphere DAN regions (left FEF and IPS) was associated with better learning (improvement of RTs over time) in the VS task. Furthermore, stronger inter-hemispheric DAN-FC (bilateral FEF) predicted better learning (RT improvement) in the Posner task but counterintuitively it was also correlated with slower (not faster) RTs in the VS task. There was no significant correlation between DAN-FC and behavioral performance in the Landmark task. However, this task appeared to be too demanding with high ERs (about 40% on average) and without significant improvement (learning) over the runs. Thus, this behavioral parameter might not have been an optimal candidate for FC-behavior correlations.

As mentioned above, the correlations between PRE-task DAN-FC and behavioral parameters in VS and Posner were not unidirectional (same accounted for the POST task FC results), i.e., stronger DAN-FC was often – but not always – associated with better performance (faster mean RT or greater improvement over the runs). One reason may be that the FC between brain regions indicates a functional connection but the direction/type of influence cannot directly be inferred (Friston, 2011), being either beneficial (excitatory) or disturbing (inhibitory). Hence, an increase of FC between two connected brain areas, in which one region functionally inhibits the other, may result in worse behavioral output, whereas the FC increase between functionally synergistic regions can lead to better performance.

Despite some dissociations, our findings principally support the previous proposal of RSNs representing individual traits that may determine the personal cognitive ability of humans (Harmelech and Malach, 2013; Sadaghiani and Kleinschmidt, 2013). Our results underline that such a relation is also found for the DAN, which is recruited during behavioral engagement in the related functional domain (visuospatial attention). Different previous studies on the relation of pre-task FC and attentional performance stressed the importance of *between-networks* FC, specifically the functional interaction of prefrontal DAN areas and visual cortices (Baldassarre et al., 2012), of right parietal DAN regions and dorsal anterior cingulate cortex belonging to the default mode network (Bueicheku et al., 2020) or between parietal DAN regions and remote subcortical/medial-temporal/orbito-frontal regions belonging to several different RSNs (Chou et al., 2013). In our study, the *within-network* FC of the DAN was shown to be predictive of the individual behavioral performance in spatial attention tasks. This confirms previous findings where performance in a visual signal detection task was related to pre-task FC between parietal and dorsolateral prefrontal DAN regions (Berry et al., 2017) and that attentional performance of children between 4 and 7 years was correlated to the individual FC in fronto-parietal DAN regions (Rohr et al., 2017). Studies assessing FC by use of electroencephalography (EEG-FC) also revealed the behavioral relevance of pre-task EEG-FC in DAN regions for attentional tasks involving audio-visual stimuli (Hipp et al., 2011) or visuo-motor performance (learning of mirror drawing skills) (Manuel et al., 2018). That the ongoing “spontaneous” activity in the RSNs may even be a prerequisite for the learning of skills is supported by a recent study using magnetoencephalography during engagement in a motor task. It showed that performance improvement relied on micro-offline gains acquired during the phases of rest interspersed between bouts of motor practice (Bönstrup et al., 2019).

In summary, our findings together with those of previous studies underline the predictive value of both within- and between-network FC of the DAN for the individual ability in visuo-spatial attention.

### Within-Network Functional Connectivity of the Dorsal Attention Network Is Stable – And Still It Seems to Adapt Following Task Performance

As revealed by ROI-to-whole brain FC analyses, the predefined DAN core regions (bilateral IPS and FEF) were functionally connected with each other as well as with the bilateral MFG and MT+, regions that are usually considered to build the DAN (Fox et al., 2006; He et al., 2007; Hacker et al., 2013). These brain regions are also recruited during active engagement in tasks that require shifts of spatial attention (Corbetta and Shulman, 2002). The ROI-to-whole brain FC as well as the ROI-to-ROI FC did not change from the PRE- to the POST-task recording. These findings are in line with previous studies reporting the DAN to be an inter-individually reproducible (Fox et al., 2006) and intra-individually highly stable RSN (Choe et al., 2015; Finn et al., 2015). The within-network FC of the DAN remains even stable when its state changes from rest to action, as previously shown in a study that compared resting-state FC to intra-task FC while participants performed a visuospatial attention task (Spadone et al., 2015).

That there was no significant change of absolute FC values from PRE to POST in our study does not mean that the DAN is not malleable or influenceable by experience. Of note, there was a certain change in the DAN-FC from PRE to POST in that several significant FC-behavior relations were only present in the PRE but not the POST rs-fMRI session and vice versa.

Hence, there must have been some kind of reorganization of the DAN’s FC between PRE and POST, either (I) in direct response to the task performance or (II) spontaneously in terms of state fluctuations. As an example, the FC-behavior relation of right FEF-IPS FC and the mean RT in the VS task was significant only in the POST session after task engagement, because only then (but not before) a faster search was associated with stronger intra-hemispheric connectivity in the right DAN. Following hypothesis I, this may indicate an early and specific adaptation of the DAN to the task demands. That a behavioral intervention (i.e., one session of task performance or a several-day cognitive training) can in principle change RSNs was previously shown for different cognitive domains. For instance, one session of a new language task was able to change the FC in the language network immediately after the task (Waites et al., 2005). A 6-weeks mnemonic training in naïve subjects induced persistent FC changes in and between the medial temporal network, DMN, and other RSNs, finally resembling those of memory athletes (Dresler et al., 2017). Regarding the DAN and visuo-spatial attention, an intense training over several days on a shape-identification task led to changes in FC between visual cortex and frontal-parietal DAN areas, which were correlated with learning effects (Lewis et al., 2009). Engaging in a visual classification task (faces or scenes) caused differential coupling between ventral frontal cortex and category-preferential visual cortex regions in subsequent periods of rest (Stevens et al., 2010). Furthermore, one session of a VS task was shown to increase the FC between the right posterior parietal cortex and the dorsolateral prefrontal cortex (Bueicheku et al., 2019). A visuo-perceptional task training (motion coherence discrimination) was shown to increase the FC of MT+ as the cortical region responsible for the processing of moving visual stimuli (Sarabi et al., 2018) but also the FC of the hippocampus as a region involved in the consolidation of early learning (Urner et al., 2013).

In the light of these studies, our findings of different FC-behavior relations in the DAN before and after performance of spatial attention tasks could be interpreted in support of task-induced DAN-FC changes. This would underline the hypothesis that the DAN – although intra-individually very stable – can be shaped by learning experiences and that RSNs may serve as a flexible, continuously updated “memory system” that helps the individual to be better prepared for upcoming cognitive challenges (Sadaghiani and Kleinschmidt, 2013; Manuel et al., 2018). It is also in line with the hypothesis that RSNs may – at least partly – be the result of learning, i.e., repetitions of task-related co-activations of different brain regions (Miall and Robertson, 2006; Harmelech and Malach, 2013; Guerra-Carrillo et al., 2014), and that this rather flexible system plays on the rigid backbone of structural connections in the brain (Sadaghiani and Kleinschmidt, 2013).

However, following hypothesis II, there is also an alternative explanation for the observed differences between the two rs-fMRI sessions (PRE/POST) regarding different FC-behavior correlations in our study: spontaneous state fluctuations over time. Previous studies accordingly emphasized the “intrinsic activity,” “ongoing dynamics,” or “time-varying FC” of RSNs fluctuating over seconds and minutes independent from specific inputs or outputs (Raichle, 2015; Sadaghiani et al., 2015; Kucyi et al., 2018). Ongoing intrinsic fluctuations over large-scale networks were also shown to determine different attentional states, such as in sustained attention and task-unrelated mind wandering (Yamashita et al., 2021; Zuberer et al., 2021). The extent of these fluctuations can still influence the upcoming behavioral performance in a cognitive task explaining inter-trial (and indirectly also inter-subject) variability (Coste et al., 2011). Interestingly, when correlating the change in FC from PRE to POST with behavioral performance parameters in our cohort, smaller DAN-FC changes were associated with larger improvement in the Posner task. Hence, one could speculate that participants with a very stable “less fluctuating” DAN had better learning in this specific task. This assumption is also supported by a previous study, which showed that task-related reduction of FC variability was associated with improved behavioral performance in a letter recognition task (Elton and Gao, 2015).

Due to the design of our study, we cannot exclude that small spontaneous fluctuations of DAN-FC over time finally led to different FC-behavior correlations in the POST than in the PRE rs-fMRI session. Task-independent spontaneous state changes could even resolve some of the discrepancies in our FC-behavior correlations, for instance, that stronger post-task FC between right FEF and IPS was related to shorter RTs in VS and at the same time to smaller RT improvement in the Posner task.

### Limitations

To thoroughly disentangle task-induced changes from time-varying spontaneous fluctuations of the RSN one would have required a control rs-fMRI experiment without an interleaved task performance, which was not part of this study. The modest sample size of this study might have prevented to detect smaller effects of PRE/POST FC changes due to a lack of statistical power. Furthermore, significant FC-behavior correlations in a rather small sample may be statistically inflated and may not be replicated in larger (>2000) brain-wide association studies (Marek et al., 2020). This also casts some doubt on the general assumption that rs-fMRI FC strongly indexes inter-individual differences in cognitive ability, as long as it is not replicated in larger brain-wide association studies.

## Conclusion

In our study, the DAN was confirmed to be an intra- and inter-individually stable RSN. The significant association between the DAN’s within-network FC and individual behavioral performance in spatial attention tasks underlines its functional relevance and gives rise to the importance of RSNs for individual cognitive ability. Although time-varying spontaneous fluctuations of DAN-FC cannot be fully ruled out as a confounder, the change of the relation between DAN-FC and behavioral performance measures following task performance support the concept of RSNs as a flexible internal memory system continuously shaped by learning experiences, helping individuals to meet upcoming cognitive challenges with improved performance.

## Data Availability Statement

The raw data supporting the conclusions of this article will be made available by the authors upon reasonable request.

## Ethics Statement

This study was reviewed and approved by the Ethics Committee of the University of Lübeck (14-189). All subjects provided their written informed consent to participate in this study.

## Author Contributions

BM: conceptualization, methodology, data analysis, visualization, and writing – original draft. LB and JI: investigation and project administration. PK: visualization and software. TM: conceptualization, resources, and writing – review and editing. CH: writing – review and editing and supervision. AS: conceptualization, methodology, visualization, software, data analysis, and writing – review and editing. All authors contributed to the article and approved the submitted version.

## Conflict of Interest

The authors declare that the research was conducted in the absence of any commercial or financial relationships that could be construed as a potential conflict of interest.

## Publisher’s Note

All claims expressed in this article are solely those of the authors and do not necessarily represent those of their affiliated organizations, or those of the publisher, the editors and the reviewers. Any product that may be evaluated in this article, or claim that may be made by its manufacturer, is not guaranteed or endorsed by the publisher.

## Footnotes

1
https://www.nitrc.org/projects/dparsf

2
http://www.fil.ion.ucl.ac.uk/spm
